# Cryotherapy as a supplementary aid to inferior alveolar nerve block in patients with symptomatic irreversible pulpitis: A randomized controlled trial

**DOI:** 10.3892/mi.2025.254

**Published:** 2025-07-22

**Authors:** Setu Katyal, Poonam Bogra, Rajinder Kumar Bansal, Vishakha Grover, Saurabh Gupta, Saru Dhir Gupta

**Affiliations:** 1 Department of Conservative Dentistry and Endodontics, I.T.S Dental College, Murad Nagar, Ghaziabad, Uttar Pradesh 201206, India; 2 Department of Conservative Dentistry and Endodontics, J N Kapoor D.A.V. Dental College and Hospital, Yamunanagar, Haryana 135001, India; 3 Department of Conservative Dentistry and Endodontics, Guru Nanak Dev Dental College and Research Institute, Sunam, Punjab 148028, India; 4 Department of Periodontics, Dr Harvansh Singh Judge Institute of Dental Sciences and Hospital, Panjab University, Chandigarh 160014, India; 5 Department of Conservative Dentistry and Endodontics, Maharishi Markandeshwar College of Dental Sciences, Ambala, Haryana 133207, India

**Keywords:** cryotherapy, endodontic, nerve block, mandible, molars, irreversible pulpitis

## Abstract

The present study aimed to evaluate the effects of cryotherapy in conjunction with inferior alveolar nerve block (IANB) on intraoperative pain and anaesthesia success in adult patients with symptomatic irreversible pulpitis (SIP) of the mandibular permanent first molars. A total of 60 patients diagnosed with SIP of the mandibular first molars were randomly divided into two groups (n=30) as follows: Group I (the control), in which patients were administered IANB with 3.6 ml 2% lignocaine, and group II (test group), in which patients received the same IANB therapy followed by cryotherapy using an endo-frost spray and intrapulpal ice sticks. Pain scores were recorded during access opening and cleaning/shaping using the Heft-Parker visual analogue scale (VAS). The primary outcome was the success rate of anaesthesia (VAS ≤54 mm). The secondary outcome was mean pain score during the treatment phase. Group II exhibited a higher success rate (79.3%) than group I (65.3%) (P=0.24). The mean pain during access opening was significantly lower in group II (20.60±29.90) than in group I (41.43±43.10) (P=0.04). Although pain scores during cleaning/shaping were lower in group II (17.73±24.06 vs. 26.95±36.72), the difference was not statistically significant (P=0.27). On the whole, the present study demonstrates that cryotherapy improved anaesthetic success and reduced intraoperative pain during access opening in patients with SIP. However, its effect during subsequent treatment stages was limited, suggesting the need for supplemental anaesthesia in some cases.

## Introduction

Achieving profound pulpal anaesthesia during endodontic treatment, particularly in mandibular molars with symptomatic irreversible pulpitis (SIP), remains a persistent clinical challenge. Inferior alveolar nerve block (IANB), although routinely employed, demonstrates a high failure rate in such cases, with reported success rates ranging from 19 to 56% owing to the heightened inflammatory state of the pulp (1,2).

Achieving effective anaesthesia in cases of SIP in the mandibular molars, particularly with an IANB, is challenging due to a combination of physiological and anatomical factors. Severe inflammation in SIP releases mediators, such as prostaglandins and bradykinin, which sensitize pulpal nociceptors, lowering the pain threshold and causing hyperalgesia. This makes the nerves hyper-responsive, reducing the efficacy of local anaesthetics (3). Additionally, the acidic environment in inflamed tissues lowers the pH level, hindering the dissociation of anaesthetics, such as lidocaine, into their active form, thus impairing nerve membrane penetration and sodium channel blockade. Inflamed nerves may also express more sodium channels, which further resisting anaesthesia (4). Anatomically, mandibular molars pose challenges owing to potential accessory innervation from nerves, such as the mylohyoid, lingual or long buccal nerves, which may not be fully blocked by a standard IANB (3). Collectively, these factors make profound anaesthesia difficult to achieve in patients with SIP.

Several strategies have been explored to improve anaesthetic outcomes, including supplemental techniques (intraosseous, intraligamentary and buccal infiltrations) and the use of different anaesthetic agents or volumes (5). While a considerable success rate has been documented for intraosseous injections, this technique not only requires specialized apparatus, but also incurs significant expenses and has the potential to inflict damage on root structures, induce systemic complications, provoke pain and result in post-injection discomfort (6). Intrapulpal injections are associated with significant pain and require pulp exposure (5).

Cryotherapy, a non-pharmacological modality involving the application of cold, is a promising adjunctive strategy. It induces local vasoconstriction, reduces oedema, reduces nerve conduction velocity and elevates the pain threshold through nociceptor desensitization (7). However, the potential risks associated with cryotherapy, such as transient tissue sensitivity or discomfort from cold application, should be considered, although these are typically minimal with controlled application (7). Previous studies have demonstrated that intraoral cryotherapy can improve the success rate of IANB, and reduce intraoperative and post-operative pain during endodontic procedures (7-10). For instance, Gopakumar *et al* (11) evaluated the anaesthetic efficacy of Endo-Ice spray and intrapulpal ice sticks as adjuncts to IANB in patients with SIP, finding that both methods significantly reduced pain and improved anaesthesia success, with ice packs exhibiting superior outcomes. Moreover, mechanistic investigations have demonstrated that cooling can suppress capsaicin-sensitive pain receptors and modulate inflammatory pathways, offering a plausible biological rationale for its analgesic effects in inflamed pulp tissues (7,8).

Although previous studies have separately investigated buccal ice packs or intrapulpal ice application as adjuncts to IANB (9,10), the combined use of surface and intrapulpal cryotherapy to maximize analgesic efficacy in patients with SIP has not yet been explored in a single randomized controlled trial (RCT), at least to the best of our knowledge. The present study aimed to address this gap by evaluating the efficacy of cryotherapy as a supplemental aid to IANB in patients with SIP undergoing endodontic treatment, with the primary outcome being the anaesthesia success rate (defined as no or mild pain during treatment), and the secondary outcome being pain scores during access opening and cleaning/shaping. By providing a non-invasive, chairside approach to enhance anaesthesia and reduce pain, cryotherapy holds significant clinical relevance for improving patient comfort and procedural outcomes in endodontic practice, particularly for challenging SIP cases. The null hypothesis was that cryotherapy would not enhance IANB efficacy compared with conventional IANB alone.

## Patients and methods

### Trial design

The present study was a single-centre, parallel-group RCT with a 1:1 allocation conducted at the Department of Conservative Dentistry and Endodontics, J N Kapoor D.A.V. Dental College and Hospital, adhering to the Consolidated Standards of Reporting Trials (CONSORT) guidelines 2025(12). The trial employed a 1:1 allocation ratio, comparing two interventions: Group I (IANB with 2% lignocaine alone) and group II (IANB with 2% lignocaine supplemented with cryotherapy). The study period spanned from April, 2023 to January, 2025. Ethical approval for the present study was obtained from the Ethics Committee of J N Kapoor D.A.V. Dental College and Hospital, Yamunanagar, India (F/EC/21/0018). The study was conducted in strict accordance with the principles outlined in the Declaration of Helsinki and in compliance with all relevant guidelines and regulations governing human research ethics. Each patient provided written informed consent prior to participation. The trial was registered in the Clinical Trials Registry of India CTRI/2023/03/050746 (https://ctri.nic.in/Clinicaltrials/pmaindet2.php?EncHid=ODEzMzU=&Enc=&userName=) (Clinicaltrials.gov). Prior to the commencement of patient recruitment, informed consent was obtained from the patients following a detailed explanation of the objectives of the study, as well as the procedures, risks and benefits. No modifications were made following trial registration.

### Selection of participants

Eligibility criteria included patients aged 18-40 years with fully erupted mandibular first molars that exhibited complete root development. The diagnosis of SIP was established based on both clinical and diagnostic findings, consistent with the American Association of Endodontists (AAE) guidelines (13). Clinically, patients presented with a history of sharp, spontaneous pain, lingering thermal sensitivity lasting >30 sec following cold stimulus, and referred pain. Diagnostic confirmation was obtained using cold testing (Endo-Frost, Coltene), in which a prolonged and exaggerated response indicated pulpal inflammation. Additionally, all included teeth exhibited positive responses to electric pulp testing (EPT), confirming pulpal vitality. Pre-operative pain intensity was required to exceed 54 mm on the Heft-Parker visual analogue scale (VAS, 170 mm scale), indicating moderate to severe pain (13). This threshold was selected based on the Heft-Parker VAS classification, where scores >54 mm correspond to moderate to severe pain, ensuring the inclusion of patients with clinically significant pain levels that challenge anaesthetic efficacy in SIP cases. This cut-off aligns with that of previous studies (14,15) and facilitates the meaningful evaluation of the effects of cryotherapy on pain management in highly symptomatic patients.

Dental anxiety was also assessed pre-operatively using Corah's Dental Anxiety Scale-Revised (DAS-R), which yields a total score ranging from 4 to 20(16). The scores were categorized as follows: ≤8, mild anxiety; 9-12, moderate anxiety; 13-14, high anxiety; and 15-20, severe anxiety or dental phobia. Only patients with DAS-R scores >9 were included to ensure a uniform level of moderate-to-high dental anxiety, as anxiety is known to lower pain thresholds and can affect the success of local anaesthesia. Including this criterion helped standardize the psychological factors affecting the anaesthetic response across participants.

The exclusion criteria included patients with systemic health conditions, a history of analgesic intake within 12 h prior to the procedure, non-vital teeth (negative to EPT), radiographic evidence of periapical pathology, such as widened periodontal ligament space or periapical radiolucency, patients with psychological or behavioural issues, a history of previous endodontic treatment and a history of chronic pain. A total of 133 patients were screened; 60 patients who met all the inclusion criteria were enrolled after obtaining written informed consent.

### Interventions and comparator

Participants were randomly assigned to two groups (n=30 teeth each) as follows: i) Group I (the control): In this group, patients received IANB with 3.6 ml 2% lignocaine hydrochloride (Xylocaine^®^) and epinephrine (1:100,000) using a standardized technique (17). The injection site was located three-quarters of the distance along an imaginary line extending from the midpoint of the coronoid notch to the deepest part of the pterygomandibular raphe. A 27-gauge needle (31 mm) was used, and the syringe barrel was positioned at the contralateral premolar or molar region. After approximately two-thirds of the needle length was advanced, and bone contact was achieved, negative aspiration was confirmed. The entire volume was slowly deposited at a rate of 1 ml/min. During needle withdrawal, a few drops were deposited to anaesthetize the lingual soft tissues and facilitate rubber dam clamp placement.

ii) Group II (experimental group): This group received the same IANB technique with a volume of 3.6 ml of 2% lignocaine and epinephrine (1:100,000) as group I. In addition, cryotherapy was administered post-anaesthesia using Endo-Frost spray (Coltene) on the occlusal, buccal and lingual surfaces for 2 sec each, followed by the intrapulpal application of sterile ice sticks (6 mm in diameter and 3 cm in length) for 4 min after access cavity preparation and pulp chamber deroofing. Sterile ice sticks were prepared and stored in a digital freezer (Gellvann). The temperature of the ice sticks was monitored using a digital freezer thermometer to ensure consistency, maintaining a range between -4 and 0˚C to prevent thermal injury. Following access cavity preparation and pulpal exposure, the ice sticks were gently placed inside the pulp chamber using sterile tweezers and maintained in contact for 4 min. Care was taken to monitor any signs of patient discomfort or tissue sensitivity during application.

Root canal treatment, including access cavity preparation, cleaning, shaping and irrigation, was standardized for both the groups. Access cavities were prepared using a high-speed airotor handpiece with Endo Access burs (Dentsply Sirona) under water cooling. Working length was determined using an electronic apex locator (Root ZX II, J. Morita) and confirmed radiographically. Cleaning and shaping were performed using ProTaper Gold rotary instruments (Dentsply Sirona) using a crown-down technique. Irrigation was performed with 3% sodium hypochlorite (Prime Dental Products Pvt. Ltd.) using side-vented irrigation needles (NaviTip, Ultradent Products Inc.), followed by a final rinse with 17% EDTA (MD-Cleanser, Meta Biomed) and distilled water.

At 20 min following IANB administration, the effectiveness of local anaesthesia was verified using both subjective and objective criteria. Subjective signs included numbness of the lower lip and tongue, confirming anaesthesia of the inferior alveolar and lingual nerves. Objective testing involved EPT (D640 Digitest II Pulp Vitality Tester, Parkell) at two intervals (2 min apart) and a cold stimulus test using a cotton pellet moistened with Green Endo-Ice spray (Coltene/Whaledent Inc.) applied for 5 sec. Only teeth that exhibited no response to either the EPT or the cold test were included. Patients who exhibited a positive response to either test were excluded from the study and received supplemental intrapulpal or intraligamentary injections. During treatment, pain was recorded using the Heft-Parker VAS, categorized as 0 mm (no pain), <54 mm (mild pain), 54-114 mm (moderate pain) and >114 mm (severe pain). Successful anaesthesia was defined as no or mild pain (VAS ≤ 54 mm) during access and cleaning/shaping. Moderate to severe pain was considered a failure, and such cases were managed with supplemental anaesthesia.

### Outcomes

A total of 4 patients in the control group and patient in the experimental group withdrew during follow-up due to failure to anaesthesia. Thus, 26 patients in the control and 29 patients in the experimental group completed all study visits (T1-T3) and were included in the final analysis. The primary outcome was the success rate of anaesthesia, defined as the percentage of patients reporting no to mild pain (≤54 mm on the Heft-Parker VAS) during access opening and cleaning/shaping. The secondary outcome was the pain score recorded on the Heft-Parker VAS during these procedures.

### Data management and harms

The data were stored in a secure password-protected database with anonymized participant IDs. Only the principal investigator and statistician accessed the final data set. For the present study, one interim analysis was conducted at 50% enrolment to assess the safety and preliminary efficacy, as planned in the protocol. The Data Monitoring Committee (DMC), J N Kapoor D.A.V. Dental College and Hospital, reviewed the primary endpoint (adverse event rate) using a Haybittle-Peto boundary (P<0.001) to control for type I errors. No significant harm was observed (P>0.001 for severe adverse events), and the futility criteria (conditional power <20%) were not met, allowing the study to continue to completion as planned.

### Sample size estimation

The sample size was determined using G*Power software (version 3.1.9.2, Heinrich-Heine-Universität Düsseldorf, Germany) with an alpha error of 5% and statistical power of 80%. Based on an effect size of 0.70 from a previous study (14), and a projected attrition rate of 10% in each group, 60 patients (30 per group) were deemed sufficient to detect a clinically significant difference in anaesthesia success rates.

### Randomization

The personnel who enrolled participants and those who assigned them to the interventions did not have access to the random allocation sequence. Patients were randomly divided into the experimental or control group using a computer-generated sequence (random.org) with a block randomization method (block size of four) to ensure balanced allocation. The principal investigator was blinded to the randomization process to prevent bias, and allocation concealment was maintained using sequentially numbered opaque sealed envelopes that were opened only on the day of the procedure.

### Blinding

The blinding of the operator and participants was not feasible due to the nature of cryotherapy intervention. However, the outcome assessor responsible for recording the Heft-Parker VAS scores was blinded to the group allocation to reduce bias.

### Statistical analysis

An intention-to-treat (ITT) analysis was performed to account for dropouts; however, primary results are reported for per-protocol completers. Data were entered into a Microsoft Excel spreadsheet and analysed using Stata Statistical Software Release 18 (StataCorp, LP). The normality of the data distribution was assessed using the Shapiro-Wilk test, with confirmation via Q-Q plots. The data were found to be normally distributed. Data were summarized using descriptive statistics: quantitative variables (e.g., Heft-Parker VAS scores) and are reported as the mean ± standard deviation (SD), and qualitative variables (e.g., success rates) are presented as numbers and percentages. The intergroup comparisons of pain score were performed using an independent t-test and intragroup comparisons of the mean pain score were performed using repeated measures ANOVA with the Bonferroni post hoc test. The chi-square test was used to compare anaesthesia success rates between the groups, with a significance level of 0.05. All analyses followed the intention-to-treat principle and included all randomized participants in the final analysis.

## Results

**Figure 1:**
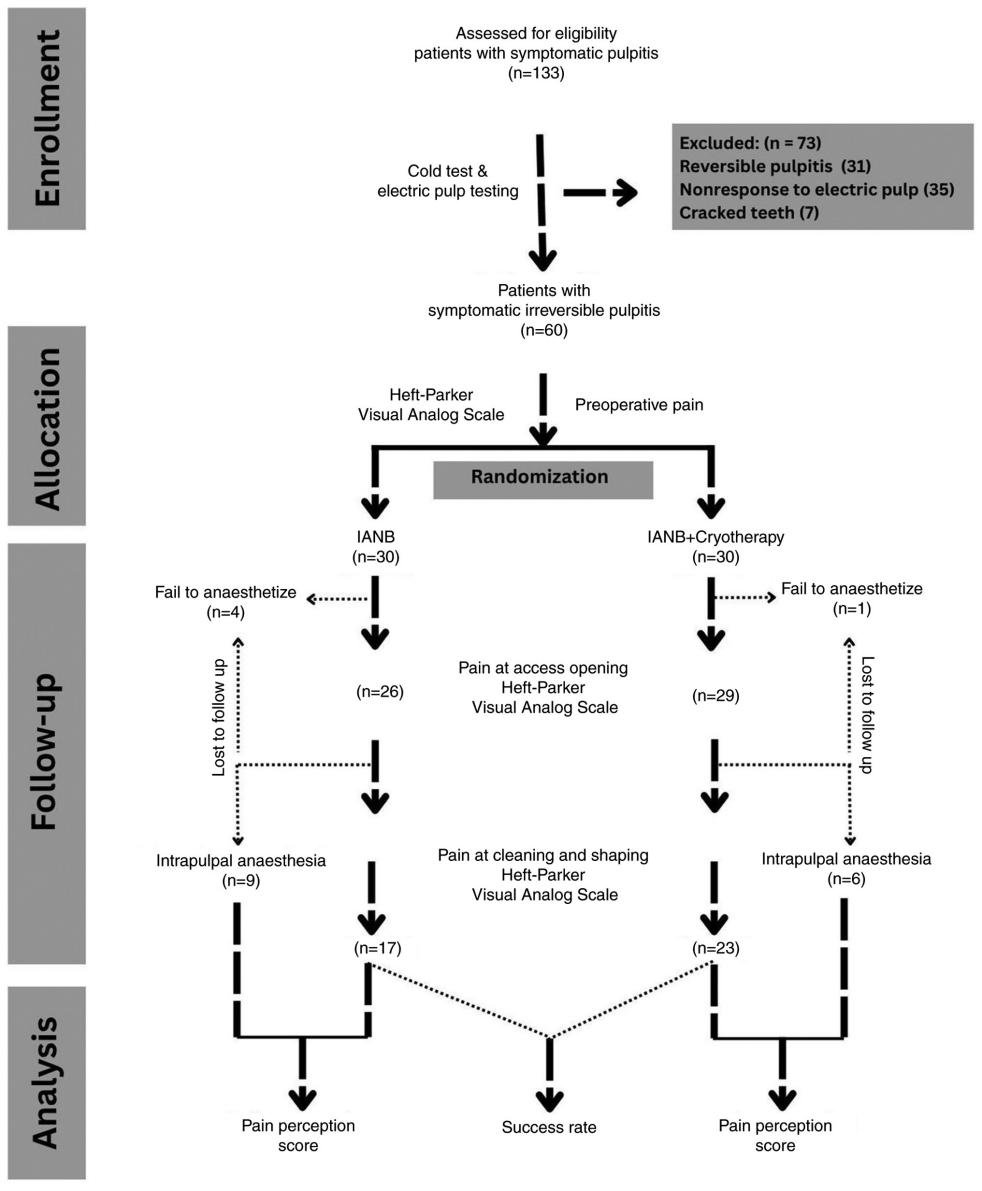
CONSORT flowchart.

The CONSORT flow diagram is presented in Fig. 1. The study results demonstrated the baseline comparability (Table I) of group I and group II, with the mean ages of the patients being 32.8±6.20 and 32.6±6.31 years, respectively (P=0.9), and a non-significant sex distribution, with each group consisting of 15 males (25%) and 15 females (25%).

The pre-operative pain scores were similar between the groups (99.17±15.57 vs. 95.33±18.56, P=0.39) (Table II). This demonstrated that both groups were comparable at baseline. Group II exhibited significantly lower pain during access opening compared with group I (20.60±29.90 vs. 41.43±43.10, P=0.03), although not during cleaning and shaping (17.73±24.06 vs. 26.95±36.72, P=0.27) (Table II). Intragroup analysis via repeated measures ANOVA confirmed a significant pain reduction over time in both groups (Table III). Post hoc analysis with the Bonferroni test revealed significant pain reductions from preoperative to access opening and cleaning/shaping in both groups (P=0.0003), with greater reductions in group II (Table IV), but no notable difference between cleaning/shaping and later stages. The overall anaesthesia success rate was higher in group II (79.3%, 23/29) than in group I (65.3%, 17/26) (P=0.247), indicating that cryotherapy enhanced IANB efficacy in patients with SIP; however, this difference between the groups was not significant (Table V).

## Discussion

Effective pain management remains the cornerstone of successful endodontic therapy, with the ability to achieve profound anaesthesia, often serving as a critical measure of clinical competence. IANB is the gold standard for anesthetizing mandibular teeth during endodontic procedures. However, achieving adequate anaesthesia in teeth with SIP presents a significant challenge owing to the acute inflammatory state of the pulp. Inflammation in SIP leads to an increased expression of tetrodotoxin-resistant sodium channels on nociceptors, which are less responsive to local anaesthetics, reducing the success rate of IANB by up to eight-fold (18). This physiological barrier, combined with other factors, such as patient anxiety, inaccurate injection techniques and anatomical variations, has prompted the exploration of supplementary methods to enhance the efficacy of IANB (5). Despite these efforts, no technique has achieved complete pulpal analgesia, underscoring the need for innovative approaches to improve pain control during endodontic treatments.

The present study utilized a volume of 3.6 ml of 2% lidocaine for IANB, based on the findings presented in the study by Aggarwal *et al* (19), who reported a higher success rate with this dosage compared to 1.8 ml in patients with SIP and reported similar success rates with 2% lidocaine, 4% articaine and 0.5% bupivacaine. This choice of anaesthetic volume aligns with the aim of the study of optimizing the efficacy of IANB as a baseline for comparison with cryotherapy. Cryotherapy was administered to the experimental group (group II) using a combination of refrigerant spray (Endo-Frost, Coltene) and intrapulpal ice sticks. Cryotherapy has been explored in endodontics in various forms, including cold saline irrigation and intraoral ice packs, with evidence suggesting that it can mitigate postoperative pain and enhance anaesthetic outcomes (9,20). For instance, it has been demonstrated that cold saline irrigation significantly reduces postoperative pain in teeth with vital pulps, likely by reducing inflammation and pulpal nerve activity (21).

In the present study, group II (cryotherapy) demonstrated significantly lower mean pain scores than group I (control) during access opening (P<0.001), indicating that cryotherapy effectively enhanced the efficacy of IANB at this stage of treatment. However, during cleaning and shaping, while group II exhibited a lower mean pain score, the difference was not statistically significant (P>0.05). This non-significant difference may be attributed to the transient nature of the analgesic effects of cryotherapy, as the cooling-induced nociceptor desensitization and reduced pulpal blood flow likely diminish over time (7). The intrapulpal ice application, administered for 4 min immediately following access cavity preparation, may have exerted its maximum effect during the initial stages of treatment, with the cryoanesthetic effect waning by the time cleaning and shaping commenced, typically 10-15 min later in the procedure. This temporal limitation is consistent with prior studies, such as Vera *et al* (22), which noted that the effects of cryotherapy are most pronounced within a short window post-application due to tissue rewarming and restoration of normal neural conduction. Additionally, the mechanical stimulation and irrigation during cleaning and shaping may further activate sensitized nociceptors in inflamed pulp tissue, potentially counteracting the residual cryotherapy effect (21). These factors likely explain the lack of a significant difference at this later stage, suggesting that supplemental cryotherapy applications or alternative adjunctive techniques may be necessary to maintain analgesia throughout the entire endodontic procedure.

The present study experienced a higher dropout rate in the control group (4 patients) compared with the experimental group (1 patient), primarily due to anaesthesia failure, raising the possibility of skewed outcomes. However, the use of an ITT analysis, alongside the primary per-protocol analysis of completers (26 control and 29 experimental patients), helped mitigate potential bias by including all randomized participants in the final analysis.

Cryotherapy induces vasoconstriction and diminishes cellular metabolism by restricting biochemical processes, which reduces the extent of tissue injury, consequently reducing the oxygen requirements of cells and curtailing the production of free radicals within tissues. The vasoconstrictive response yields anti-edematous effects, whereas analgesia is attained following a decrease in temperature due to the inhibition of nerve endings resulting from the application of cold stimuli (7). The magnitude of the vasoconstriction reaches its peak at a temperature of 15˚C, and research has indicated that a reduction in body temperature diminishes peripheral nerve conduction; notably, at ~7˚C, there is a total deactivation of myelinated A delta fibres, whereas the non-myelinated C-fibres become inactive at ~3˚C (23).

Overall, in the present study, group II achieved a higher success rate of anaesthesia than group I, with a statistically significant difference. These findings are consistent with those of Topçuoğlu *et al* (24), who reported that preoperative intraoral ice pack application for 5 min significantly improved IANB success rates in patients with SIP. Similarly, Gopakumar *et al* (11) evaluated the effect of intraoral cryotherapy using ice sticks and refrigerant spray, and found that both methods significantly reduced pain and improved IANB outcomes in SIP cases, with ice packs showing superior results, as observed in the present study.

The application of Endo-Frost refrigerant spray (-50˚C), composed of propane, butane and isobutane, in combination with intrapulpal ice sticks for 4 min, likely contributed to the observed pain reduction in group II. Vera *et al* (22) suggested that the optimal duration of cryotherapy varies by tissue type, with 4-5 min being sufficient to achieve therapeutic effects without causing damage in areas with minimal muscle and fat, as in intraoral applications. The 4-min duration for intrapulpal ice application used in the present study was selected based on this evidence, as it balances the need for effective nociceptor desensitization and reduced pulpal blood flow with the prevention of thermal injury to pulpal or surrounding tissues. This duration is further supported by practical considerations, as preliminary testing indicated that 4 min allowed consistent cooling of the pulp chamber without patient discomfort or procedural delays, aligning with findings from Gopakumar *et al* (11), who used a similar duration for intrapulpal ice application. By contrast, Koteeswaran *et al* (25) found no significant difference between IANB alone and IANB with cryotherapy using Endo-Ice spray (-26.2˚C) and intrapulpal ice. This discrepancy may be attributed to the lower temperature of Endo-Frost (-50˚C) used in the present study, which likely produced a more pronounced cryoanesthetic effect compared to Endo-Ice, facilitating deeper tissue cooling and greater pain reduction.

The physiological mechanisms of cryotherapy provide the rationale for its effectiveness. Cold application extracts heat from the tissue, causing vasoconstriction and reducing local inflammation, thereby mitigating oedema and inflammation (7). Goodis *et al* (26) reported that tooth cooling decreased pulpal blood flow, which can eliminate pain perception in some cases. Additionally, cryotherapy reduces the adherence of leukocytes to capillary endothelial walls and decreases endothelial dysfunction (7). According to Van't Hoff's law, a 10˚C decrease in tissue temperature reduces local enzyme activity and cellular metabolism by 2-3-fold, minimizing tissue damage (7). Furthermore, cryotherapy disrupts nerve conduction by causing myelin sheath deterioration and axonal degeneration, raising the nociceptive threshold, and slowing pain signal transmission (19). Cold also inactivates oral vanilloid receptors, which are upregulated during inflammation and are sensitive to capsaicin, further reducing pain perception (27). These mechanisms likely explain why patients often use cold (e.g., ice or cold water) to alleviate acute pulpal pain, a clinical observation supported by prior literature (28,29). Collectively, these factors likely contributed to the significant pain reduction observed in group II compared with group I during endodontic treatment.

Demographic variables (pre-operative pain, age and sex) were comparable between the two groups, indicating that these factors did not influence the study outcomes. Consequently, the null hypothesis that there would be no difference in IANB efficacy between group I and group II was rejected. The superior performance of the cryotherapy group underscores its potential as a non-invasive, chairside technique to enhance IANB efficacy in SIP cases.

Previous studies evaluating cryotherapy as an adjunct to IANB in patients with SIP have provided valuable insight, but are not without potential biases (9,11,15). A number of studies, including those examining buccal ice packs or intrapulpal ice application, often faced challenges with blinding due to the sensory nature of cold application, which may introduce placebo effects or patient reporting bias, particularly when subjective pain scales like the Heft-Parker VAS were used (11,30). Additionally, small sample sizes in some trials may have limited statistical power, potentially overestimating or underestimating the efficacy of cryotherapy. Variability in patient selection criteria, such as inconsistent preoperative pain thresholds or inclusion of patients with differing levels of dental anxiety, could further confound results, as these factors influence pain perception and anaesthesia outcomes (15,31). These methodological limitations highlight the need for more robust study designs, such as larger sample sizes and the use of sham cryotherapy or objective pain assessment methods, to minimize bias and enhance the reliability of findings in future research.

The findings of the present study suggest several clinical implications for integrating cryotherapy into the management of SIP in endodontic practice. First, cryotherapy, using a combination of cold spray and intrapulpal ice application, can serve as an effective adjunct to inferior alveolar nerve block anaesthesia, particularly for patients with severe pre-operative pain. This approach enhances patient comfort during initial treatment stages, potentially improving procedural efficiency and patient cooperation in challenging cases. Second, in settings where immediate root canal therapy is not available, such as emergency departments or rural clinics, cryotherapy could provide temporary pain relief by reducing pulpal inflammation and nerve sensitivity, acting as a bridge to definitive treatment while referrals are arranged. Third, by decreasing inflammation and nerve responsiveness, cryotherapy may help reduce post-operative pain, potentially lowering the need for analgesics and supporting a smoother recovery. Incorporating cryotherapy into standard protocols for managing symptomatic irreversible pulpitis offers a non-invasive, cost-effective strategy to improve anaesthesia outcomes and patient experience. However, further research is required to establish standardized protocols, including optimal timing and frequency of cryotherapy application, to ensure consistent benefits across diverse clinical settings.

Despite these promising results, the present study has several limitations. The small sample size (n=60) may limit the generalizability of the findings, necessitating larger trials to confirm the efficacy of cryotherapy. Additionally, the lack of blinding for both operators and patients due to the nature of cryotherapy application introduces a potential for bias, which could have influenced pain reporting. Future studies could mitigate this limitation by implementing sham cryotherapy, such as using a non-cooled spray or room-temperature sticks, to blind participants and operators while maintaining the procedural appearance of cryotherapy application. Additionally, employing objective pain assessment methods, such as pulpal nerve stimulation or physiological monitoring (such as heart rate variability), could further reduce bias in pain reporting. Variations in operator skill during cryotherapy application or differences in pulp chamber anatomy across patients may have also influenced the consistency of the effects of cryotherapy, as these factors could affect the precision of ice stick placement or the extent of cooling achieved. These strategies would enhance the robustness of future trials evaluating the efficacy of cryotherapy in endodontic pain management.

In conclusion, cryotherapy, as a supplementary technique, significantly enhances the efficacy of IANB in patients with SIP, particularly during access opening, offering a simple and non-invasive method to reduce pain and improve patient comfort. The higher anaesthesia success rate compared to conventional IANB alone underscores its potential as a valuable adjunct in endodontic practice. However, larger, well-controlled studies are required to validate these findings and address the limitations of the present study, ensuring the consistent integration of cryotherapy into standard protocols for managing SIP.

## Figures and Tables

**Table I:** Descriptive analysis of the study groups.

Parameters	Group I	Group II	Statistical value	P-value
Age (years), mean ± SD	32.8±6.20	32.6±6.31	0.123	NS^a^
Male, n (%)	15 (25%)	15 (25%)	0	NS^b^
Female, n (%)	15 (25%)	15 (25%)		

^a^P-value >0.05; NS, non-significant difference determined using an independent t-test;

^b^P-value >0.05; NS, non-significant difference determined using the Chi-squared test. SD, standard deviation; Group I (control), patients administered IANB with 3.6 ml of 2% lignocaine; group II (test group), patients received the same IANB followed by cryotherapy using an endo-frost spray and intrapulpal ice sticks; IANB, inferior alveolar nerve block.

**Table II:** Intergroup comparison of Heft-Parker VAS pain scores at different time intervals using an independent t-test.

Pain	Group	No. of patients	Mean ± SD	Stats	P-value
Pre-operative (T0)	Group I	30	99.17±15.57	0.86	0.39
	Group II	30	95.33±18.56		
Access opening (T1)	Group I	26	41.43±43.10	2.10	0.04^a^
	Group II	29	20.60±29.90		
Cleaning and shaping (T2)	Group I	26	26.95±36.72	1.11	0.27
	Group II	29	17.73±24.06		

^a^P-value <0.05, significant difference. Data are presented as the mean ± SD. SD, standard deviation; Group I (control), patients administered IANB with 3.6 ml of 2% lignocaine; group II (test group), patients received the same IANB followed by cryotherapy using an endo-frost spray and intrapulpal ice sticks; IANB, inferior alveolar nerve block.

**Table III:** Intragroup comparison of Heft-Parker VAS pain scores at different time intervals (T0, T1 and T2) using repeated measures ANOVA.

Groups	Pre-operative (T0)	Access opening (T1)	Cleaning and shaping (T2)	Statistical value	P-value
Group I	99.17±15.57	41.43±43.10	26.95±36.72	38.07	0.001^a^
Group II	95.33±18.56	20.60±29.90	17.73±24.06	95.8	0.001^a^

^a^P-value <0.05, significant difference. Data are presented as the mean ± SD. SD, standard deviation; Group I (control), patients administered IANB with 3.6 ml of 2% lignocaine; group II (test group), patients received the same IANB followed by cryotherapy using an endo-frost spray and intrapulpal ice sticks; IANB, inferior alveolar nerve block.

**Table IV:** Post-hoc analysis with the Bonferroni test for pairwise comparison of Heft-Parker VAS pain scores at three different time intervals (T0, T1 and T2) in both the groups.

	Group I			Group II		
Pairwise group comparison	Mean difference	95% CI	P-value	Mean difference	95% CI	P-value
T0 vs. T1	57.74	-78.61 to -36.86	0.0003^a^	74.73	-89.88 to -59.57	0.0003^a^
T0 vs. T2	72.22	-93.09 to -51.34	0.0003^a^	77.60	-92.75 to -62.44	0.0003^a^
T1 vs. T2	14.48	-35.35 to 6.39	0.6864	2.87	-18.023 to 12.28	0.9989

^a^P-value <0.05, significant difference; significant at 95% confidence interval (CI). Group I (control), patients administered IANB with 3.6 ml of 2% lignocaine; group II (test group), patients received the same IANB followed by cryotherapy using an endo-frost spray and intrapulpal ice sticks; IANB, inferior alveolar nerve block.

**Table V:** Overall success rate of anaesthesia in both the groups.

Parameters	Group I (n=26)	Group II (n=29)	Chi-squared test	P-value
Successful anaesthesia	17 (65.3%)	23 (79.3%)	1.34	0.247^a^
Unsuccessful anaesthesia requiring supplemental intrapulpal/intraligamentary injections	09 (34.7%)	06 (20.7%)		

^a^P-value >0.05, no significant differences.

## Data Availability

The data generated in the present study may be requested from the corresponding author. The present randomized controlled trial is registered as.
